# Transperitoneal vs. extraperitoneal radical cystectomy for bladder cancer: A retrospective study

**DOI:** 10.1590/S1677-5538.IBJU.2017.0441

**Published:** 2018

**Authors:** Jagdeesh N. Kulkarni, Himanshu Agarwal

**Affiliations:** 1 Department of Urology, Bombay Hospital and Medical Research Centre, Mumbai, India

**Keywords:** Urinary Bladder Neoplasms, Cystectomy, Retrospective Studies

## Abstract

**Purpose:**

Conventional transperitoneal radical cystectomy (TPRC) is the standard approach for muscle invasive bladder cancer. But, the procedure is associated with significant morbidities like urinary leak, ileus, and infection. To reduce these morbidities, the technique of extraperitoneal radical cystectomy (EPRC) was described by us in 1999. We compared these two approaches and the data accrued forms the basis of this report.

**Materials and Methods:**

All patients who underwent radical cystectomy for bladder cancer by the author (JNK) with follow-up for at least 5 years were included. A total of 338 patients were studied, with 180 patients in EPRC group and 158 in TPRC group.

**Results:**

There were 3 mortalities within 30 days in TPRC group and one in EPRC group. Early complication rate was 52% and 58% in EPRC and TPRC groups. Urinary leak occurred in 31 (9.2%) patients (13 in EPRC, 18 in TPRC, p=0.19). Gastrointestinal complications like ileus occurred in 9 (5%) patients in EPRC group and in 25 (15.8%) patients in TPRC group, (p<0.001). Wound dehiscence occurred in 29, and 36 patients in EPRC and TPRC groups respectively. The reoperation rate was 6.1% and 12% in EPRC and TPRC groups, (p=0.08). Intestinal obstruction were significantly less in EPRC group (1.7% vs. 7.8% in TPRC group, p=0.002). Uretero-enteric anastomosis stricture was seen in 10 patients (4 in EPRC, 6 in TPRC, p=0.39).

**Conclusions:**

The EPRC is associated with decrease gastrointestinal complications, ease of management of urinary leaks, and low reoperation rates. Thus EPRC appears safe functionally and oncologically.

## INTRODUCTION

The Transperitoneal Radical Cystectomy (TPRC) is an established procedure that involves antegrade bladder mobilization and dissection followed by intraperitoneal placement of a neobladder or an ileal conduit. Mortality rate of Radical Cystectomy (RC) has reached a plateau (1-3%) (1) over the last two decades. However, the morbidity of RC continues to be significant, ranging from 18-30% (2), and includes ileus, fascia dehiscence, and urine leak. Furthermore, the early and late reoperation rates have reduced to 5% and 10% respectively (3). With the aim of reducing the morbidity, the technique of extraperitoneal radical cystectomy (EPRC) was described by us in 1999 (4). The outcome data of morbidity and pathological findings in both groups (EPRC & TPRC) forms the basis of this retrospective study.

## MATERIALS AND METHODS

All patients who underwent RC for urothelial bladder cancer by the senior author from January 1999 to December 2009 with a minimum of 5 years of follow-up were included. Patients were alternatively assigned to EPRC and TPRC group. Patients with bulky tumors involving the bladder neck, seminal vesicle, and Denonviller's fascia or undergoing cystectomy in the salvage setting were deemed unfit for EPRC and were hence excluded from our study. The Charlson comorbidity index (5) (CCI) was used to quantify comorbidity. This study compared TPRC and EPRC procedures with regard to clinical and pathological characteristics, operating time, blood loss, perioperative complications, 30 day mortality, and oncological outcome at 5 years. The study was approved by the institutional ethical committee. Informed consent was obtained from all persons prior to their inclusion in the study.

EPRC was performed as per our technique described in 1999 (4). The transperitoneal surgical technique was performed in the usual manner as described by Hautmann (6). The standard pelvic lymph node dissection was performed in the same manner and extension as described in the extraperitoneal surgical technique. In both groups, patients were started on liquid diet and full bowel preparation with Peglac^TM^ was given to all patients. Perioperative antibiotics were administered in all patients. All patients were followed up in our multidisciplinary ICU and managed as per institutional protocols. Feeding was started with resumption of bowel sounds and gradually escalated as per patient tolerance. Abdominal drains were removed when drainage became insignificant (<50mL). All occurrences requiring medication or a surgical intervention were assessed as a complication. Post-operative ileus is defined as postoperative nausea or vomiting associated with abdominal distension requiring cessation of oral intake and intravenous fluid support and/or nasogastric tube placement. Ileus also included intolerance of oral intake by postoperative day 5, resulting in fasting with or without nasogastric tube placement or antiemetic medication administration. The data were collected based on the detailed clinical and outpatient follow-up. Patients were seen at 3-month intervals during the first year after surgery, half yearly during the next 2 years and then once a year.

Statistical calculations were performed with SPSS 22.0 for Windows. Analysis was done according to data scaling using the Mann--Whitney U test, Chi-square test or, Unpaired t test. p values below 0.05 were considered statistically significant.

## RESULTS

During the study period, 378 patients underwent RC; however, only 338 patients were analyzed. Of them, 180 and 158 patients had classic EPRC and TPRC respectively. Demographic data including age range, comorbidity status, prior abdominal surgery, and clinical stage of the disease were comparable in the two groups (Table-1 and Table-2).

**Table 1:** Patients’ clinical characteristics.

Characteristics		EPRC group (N=180)	TPRC group (N=158)	p value
		n (%)	n (%)	(Chi square test)
RC with ileal conduit (n)		123 (68.3%)	109 (69.0%)	0.89
RC with neobladder (n)		57 (31.7%)	49 (31.0%)	0.89
Age (mean, range)		63 (57–73)	61 (54–79)	0.77
Charlson's score (median, IQR)		4 (3 -9)	4 (2 -9)	0.83^$^
Prior pelvic/abdominal radiotherapy		2 (1.1%)	1 (0.6%)	0.64
Overweight (BMI >25)		31 (17.2%)	26 (16.5%)	0.85
**Prior abdominal surgery**				
	Appendectomy	13 (7.2%)	15 (9.5%)	0.45
	Cholecystectomy	4 (2.2%)	3 (1.9%)	0.83
	Inguinal herniotomy	5 (2.8%)	9 (5.7%)	0.18

**IQR =** Inter-Quartile Range; **$ =** Mann Whitney U test

**Table 2:** Patients’ operative and follow up characteristics.

Operative details		EPRC group (*N*=180)	TPRC group (*N*=158)	p value
		*n*(%)	*n*(%)	
Operative time: RCIC (min) Mean (Range)		272.4 (210-490)	290 (216-470)	0.70**
Operative time: RCNB (min) Mean (Range)		312 (225-565)	356.4 (210-540)	0.59**
Estimated blood loss (mL) Mean (Range)		343 (210-2800)	375 (180-3100)	0.39**
Hospital stay (days) Median (IQR)		7 (6-14)	6 (6-13.5)	0.49$
**Pathological tumor stage**				
	Organ confined (T_1_, T_2_)	83 (46%)	84 (53%)	0.19*
	Non-organ confined (T_3+_)	97 (64%)	74 (47%)	0.19*
**Nodal status**				
Number of lymph nodes resected^a^ (range)		12 (2-29)	11 (4-32)	0.69**
	Lymph node negative (N_0_)	165 (91.7%)	144 (91.1%)	0.86*
	Lymph node positive (N_+_)	15 (8.3%)	14 (8.9%)	0.86*
	Lymph node density Mean (Range)^a^	13.6% (4-49%)	17.7% (6-42%)	0.58**
	Positive surgical margins	1 (0.6%)	1 (0.6%)	0.93*
**Long term follow-up**				
Duration (months) Mean (Range)		71.6 (60-83)	69.9 (62-78)	0.49**
Local recurrence		4 (2.2%)	5 (3.2%)	0.59*
Distant metastasis		13 (7.2%)	12 (7.6%)	0.89*

**RCIC =** Radical Cystectomy with Ileal Conduit; **RCNB =** Radical Cystectomy with Neobladder; **IQR =** Inter-Quartile Range

a Defined as the ratio of the number of positive lymph nodes divided by the total number of resected lymph nodes

* Chi Square test;

** Unpaired t test, $Mann Whitney U test

The mean operating time for EPRC with ileal conduit (IC) and neobladder (NB) was 272.4 min (range 210-490min) and 312.2 min (range 225-565 min) respectively. Similarly the mean operating time for TPRC with IC and NB was 290.6 (range 216-470min) and 356.4 min (range 210-540 min) respectively, (p=0.53). The mean estimated blood loss (EBL) was 343mL (range 210-2800mL) for the EPRC group and 375mL (range 180-3100mL) for the TPRC group (p=0.43). The median hospital stay was 7 (6-14) days for EPRC and 6 (6-13.5) days for TPRC groups, (p=0.49, Mann Whitney U test).

The histopathology of the RC specimen ranged from pT1 G3 pN0 M0 to pT4a pN2 M0. Pathologically, 46% patients in the EPRC group and 53% patients in the TPRC group had organ confined disease. Lymph node yield was identical in the two groups. An average yield of 12 lymph nodes (range 2-29) was seen in the EPRC and 11 (range 4-32) in the TPRC group. Fifteen patients in the EPRC approach and 14 patients in the TPRC approach had lymph node involvement. One specimen from each group had positive margins. On follow-up in the EPRC group, 4 patients developed local recurrence and 13 patients developed distant metastasis. In the TPRC group, local recurrence developed in 5 patients and 12 patients developed distant metastasis. The difference was not statistically significant.

Early complications are given in Table-3. Lone patient from EPRC group succumbed to massive pulmonary embolism. Of the 3 postoperative mortalities from TPRC group, 1 had massive myocardial infarction, while the other 2 died of septicemia following intestinal leak.

**Table 3:** Early complications.

Complication		EPRC group (*N*=180)	TPRC group (*N*=158)	p value*
		*n* (%)	*n*(%)	
30 day mortality		1 (0.6%)	3 (1.67)	0.38
Over all complication ratea		52%	58%	0.27
**Urinary complications**				
	Urine leak	13 (7.2%)	18 (11.4%)	0.19
**Site**				
	Uretero-enteric anastomosis	11	14	
	Neobladder	2	4	
**Treatment**				
	Continuous extraperitoneal drainage	8	1	
	Percutaneous nephrostomy	3	11	
	Re-exploration	2	6	
	Renal failure	23 (12.8%)	20 (12.7%)	0.97
	Pyelonephritis	2 (1.1%)	2 (1.2%)	0.89
**Gastrointestinal complications**				
	Ileus	9 (5%)	25(15.8%)	**<0.001**
	Bowel leak/Intra-abdominal abscess	2(1.1%)	4(2.5%)	0.32
**Wound complications**				
	Major dehiscence	5 (2.7%)	5 (3.1%)	0.83
	Minor dehiscence	24 (13.3%)	31 (19.6%)	0.12
**Stoma related (Ileal conduit)**				
	Stoma retraction/necrosis	2 (1.6%, N=123)	2 (1.8%, N=109)	0.90
	Peristomal excoriations	29 (23.5%, N=123)	20 (18.3%, N=109)	0.33
**Cardiac**				
	Myocardial infarction	1(0.5%)	2(1.2%)	0.49
	Arrythmia	2(1.1%)	2(1.2%)	0.89
**Pulmonary**				
	Pneumonia	7 (3.9%)	10 (6.3%)	0.30
	Respiratory insufficiency	1(0.5%)	3 (1.9 %)	0.25
**Thromboembolism**				
	Deep leg vein thrombosis	2 (1.1%)	2 (1.2%)	0.89
	Pulmonary embolism	1(0.5%)	1(0.6%)	0.93
**Neurologic**				
	CVA/TIA	1(0.5%)	1(0.6%)	0.93
**Reoperations**		**11 (6.1%)**	**19 (12%)**	**0.08**
	Urine leak	2	6	
	Bowel leak	2	4	
	Resuturing of abdominal wound	5	5	
	Stoma retraction/necrosis	2	2	

**a** = Patients with one or more complications;

* Chi square test

Broadly one or more early complications occurred in 94 (52%) patients in EPRC group and 92 (58%) patients in the TPRC group (p=0.27). A total of 31 (9.2%) patients had urinary leak, of them 13 and 18 belonged to EPRC and TPRC groups, respectively. The site of urinary leak was uretero-enteric junction in 25 patients, while remaining 6 patients had leak from neobladder suture line or urethro-neobladder anastomosis. Out of 13 patients in the EPRC group, 8 recovered with continuous extraperitoneal drainage inserted during the primary surgery, 2 patients required re-exploration and 3 required percutaneous nephrostomy (PCN). In the TPRC group, out of 18 patients, 11 had PCN, 6 had re-exploration, and lone patient recovered with continuous peritoneal drainage.

Gastrointestinal complications like ileus, was seen in 9 (5%) and 25 (15.8%) patients in EPRC and TPRC groups, respectively, p<0.001. Additional 2 and 4 patients in EPRC and TPRC group had major bowel leak or intra-abdominal abscess formation requiring re-exploration.

Wound dehiscence occurred in 29 (16.1%) patients in EPRC group and 36 (20.0%) patients in TPRC group had wound dehiscence. Five patients in each group required re-suturing. Two patients in each group had stoma retraction requiring surgical revision. Cardiac, pulmonary, and vascular complications rates did not differ between the two groups and are depicted in Table-3. The overall reoperation rate within first month of surgery was 6.1% in the EPRC and 12% in the TPRC group (p=0.08).

Table-4 describes the late complications. In the EPRC group, 36 (20%) patients had one or more late complications, as compared to 40 (25%) patients in the TPRC group, p=0.24. A total of 10 patients (4 in EPRC and 6 in TPRC, p=0.39) had significant uretero-intestinal stricture requiring endoscopic dilatation and stenting. Amongst neobladder patients, 2 in EPRC group and 1 in TPRC group (p=0.65), had urethro-neobladder stricture requiring endoscopic dilatation. The diurnal urinary incontinence rates at one year were 7.0% in EPRC and 14.3% in TPRC group, p=0.22. Nocturnal urinary incontinence was present in 24.6% patient in EPRC and 26.5% patients in TPRC group, p=0.82.

**Table 4:** Delayed complications.

Delayed complications		EPRC Group (*N*=180)	TPRC group (*N*=158)	p value*
		*n*(%)	*n*(%)	
Overall complication ratea		36 (20%)	40 (25%)	0.24
Uretero-intestinal stricture		4 (2.2%)	6 (3.8%)	0.39
Urethro-neovesical anastomotic stricture (NB)		2 (3.5%, N=57)	1 (2.0%, N=49)	0.65
Diurnal urinary incontinence (1 year, NB)		4 (7.0%, N=57)	7 (14.3%, N=49)	0.22
Nocturnal urinary incontinence (1 year, NB)		14 (24.6%, N=57)	13 (26.5%, N=49)	0.82
**Intestinal obstruction**		3 (1.7%)	14 (7.8%)	0.002
	Conservative management	3	10	
	Re-exploration	0	4	
Incisional hernia		4 (2.2%)	6 (3.8%)	0.39
Pelvic Lymphocele		5(2.8%)	1 (0.6%)	0.13
	Conservative management	3	1	
	Percutaneous (pigtail) drainage	2	0	
Metabolic acidosis (NB)		18 (31.6%, N=57)	15 (30.6%, N=49)	0.89
Reoperations		6 (3.3%)	11 (7%)	0.13
	Uretero-enteric stricture	4	6	
	Urethral anastomotic stricture	2	1	
	Intestinal obstruction	0	4	

* = Chi square test; **a =** patients with one or more complications; **NB =** Neobladder patients

Intestinal obstruction developed in 3 and 14 patients in EPRC and TPRC groups, respectively (p=0.002); of them, only 4 from TPRC group required re-exploration. Pelvic lymphocele (>100mL) was seen in 5 and 1 patients in the EPRC and TPRC group, respectively (p=0.13). Amongst the neobladder patients, 31% from each group required correction of metabolic acidosis.

## DISCUSSION

TPRC using the antegrade transperitoneal approach is the gold standard for muscle invasive bladder cancer. This approach ends up in deficient peritoneum in the pelvis and abolishes the natural compartmentalization between the gastrointestinal (GI) tract and the urinary tract, thereby resulting in bowel motility disorders in up to 25% of the patients (2, 7, 8). Hence, recently there is lot of emphasis on the re-peritonealization at the end of the cystectomy to maintain the compartmentalization of the GI tract (9).

With this concept in mind and to minimize the handling and prolonged exposure of the gut to the atmosphere, we first reported the extraperitoneal retrograde approach for RC and pelvic lymphadenectomy in 1999 (4). Secondly, our technique allowed us to close the peritoneum around the mesentery of the conduit or neo bladder, thereby maintaining the separation of the bowel anastomosis from the uretero-enteric anastomosis. Thirdly, due to late opening and early closure of the peritoneum, there was early return of the peristalsis reducing the post-operative ileus. Finally, extraperitonealization of the neobladder or conduit enabled management of urinary leaks by simple extraperitoneal drainage or transurethral catheterization alone, if necessary.

Our study clearly demonstrates the statistically significant reduction of GI complications like ileus in EPRC group (p<0.001). These results concur with the various study groups that also demonstrated similar outcomes after re-peritonealization of the intestinal tract (10, 11). Mattei et al. (12) were able to show that colonic motility is reduced in conjunction with higher creatinine concentrations in the early postoperative phase after RC and neobladder. Roth et al. (9) described a technique of creating dorsolateral peritoneal flaps to re-establish peritoneal cover. However, in our EPRC technique, the peritoneum could be closed around the mesentery of isolated bowel segment with ease.

There was no statistically significant difference between the two groups with regard to the incidence of urine leaks. However, only 38% patients from EPRC group required PCN/re-exploration in comparison to 94.4% patients in TPRC group highlighting the ease of management of post-operative urinary leaks in the EPRC group.

Our data suggest that the small infra-umbilical incision used in EPRC group and brief opening of the peritoneal cavity aids in preventing wound-related complications as originally described (4, 13). Moreover, small incision also must have helped in reducing respiratory complications in the EPRC group (4.4% vs. 8.2% in the TPRC group, p=0.15) as the upper half the abdominal wall is intact.

The low rates of intestinal obstruction observed in the EPRC group (1.7% vs. 7.8% in TPRC group, p=0.002), could be attributed to the fact that the bowel anastomosis was totally intraperitoneal without any exposure to non-peritonealized raw surface.

A slightly increased incidence of pelvic lymphocele was seen in the EPRC group (2.8% vs. 0.6% in TPRC group, p=0.13). Meticulous ligation and clipping of the lymphatics during pelvic node dissection has considerably reduced lymphocele occurrence in recent years. Similar findings have been reported by Serel et al. (10).

Late complications of the uretero-ileal anastomotic stenosis were comparable in both groups. Daytime incontinence rate at one year in ileal neobladders (n=108) were 14.3% in the TPRC and 7.0% in the EPRC group. The difference was however not significant statistically (p=0.22), possible due to low number of neobladders constructed. Minimal manipulation of rhabdosphincter, fascial attachments, and the corresponding innervations which were handled early in a bloodless field in EPRC, can be hypothesized for the better daytime continence rate in EPRC group.

Oncological safety was a genuine concern regarding the EPRC. However, our data demonstrate that the incidence of local recurrence and distant metastasis were comparable between the two groups (Table-2). The low incidence of metastasis in our study can probably be explained by the significant number of patients with pathologically node negative disease (>91% in both the groups, Table-2).

Lastly, our technique of retrograde EPRC is beneficial in terms that operability is assessed early in course of the procedure as most of the tumors are found in the trigone area. Serel et al. (10) described the technique of antegrade EPRC, in which the operability is assessed by intraoperative palpation of bladder mobility before embarking on antegrade dissection. In this technique, as the trigonal area is dealt with at the last, there is no point turning back as one is already committed to cystectomy as the ureters are divided before reaching the trigonal area.

The overall early complication rates observed in our study were relatively high. The morbidity previously reported by others varies widely from 16% to 66% (14-17). The complications published in various studies were detailed differently and are thus difficult to compare (18). Our patient population had a relatively high comorbidity with a Charlson index >3 in 42%, which partially explains the relatively high percentage of general internal complications.

Enhanced Recovery After Surgery (ERAS) protocol has been suggested recently to reduce post-operative morbidity (19) and facilitate recovery, especially in terms of early return of bowel activity and resumption of oral feeds. ERAS protocol was not followed in our patients which probably accounts for the increased duration of hospital stay. This is accepted as a fallacy of the study. EPRC facilitates this and hence ERAS protocol in these patients may be further adjunctive. However, this was not evaluated in our study.

In contemporary robotic era, it is logical to expect the possibility of robotic EPRC. However, with the current da Vinci systems, the major limiting factor is small working space in the pelvis hindering the movement of multiple robotic arms. With the future prospects of single port robots with flexible arms, robotic EPRC, although challenging, may become feasible.

This study has few limitations as it was conducted over a long period of time (1999-2009). Obviously, perioperative patient care has steadily improved over this 10-year period which might have affected our results. Owing to the non-randomized nature of our study based on comparison of two patient cohorts, further prospective randomized trials are needed to prove the real advantage of EPRC.

## CONCLUSIONS

The extraperitoneal technique (EPRC) with extraperitonealization of the neobladder or conduit is a comparatively safe and reliable surgical approach. It has penta-facta benefits in terms of reduced ileus, re-operation rates, wound problems, ease of management of urinary leaks, and improved continence rates in neobladder patients. Thus, EPRC appears safe both functionally and oncologically.
